# The distribution of bioactive gibberellins along peach annual shoots is closely associated with *PpGA20ox* and *PpGA2ox* expression profiles

**DOI:** 10.1186/s12864-022-08943-5

**Published:** 2022-10-28

**Authors:** Mengmeng Zhang, Yangtao Ma, Xianbo Zheng, Bin Tan, Xia Ye, Wei Wang, Langlang Zhang, Jidong Li, Zhiqian Li, Jun Cheng, Jiancan Feng

**Affiliations:** College of Horticulture, Henan Agricultural University, 95 Wenhua Road, Zhengzhou, Henan Province China

**Keywords:** *Prunus persica*, Gibberellin, Annual shoot, *GA20ox*, *GA3ox*, *GA2ox*

## Abstract

**Background:**

The rapid growth of annual shoots is detrimental to peach production. While gibberellin (GA) promotes the rapid growth of peach shoots, there is limited information on the identity and expression profiles of GA-metabolism genes for this species.

**Results:**

All six GA biosynthetic gene families were identified in the peach genome, and the expression profiles of these family members were determined in peach shoots. The upstream biosynthetic gene families have only one or two members (1 *CPS*, 2 *KS*s, and 1 *KO*), while the downstream gene families have multiple members (7 *KAO*s, 6 *GA20ox*s, and 5 *GA3ox*s). Between the two KS genes, *PpKS1* showed a relatively high transcript level in shoots, while *PpKS2* was undetectable. Among the seven *KAO* genes, *PpKAO2* was highly expressed in shoots, while *PpKAO1* and − 6 were weakly expressed. For the six *GA20ox* genes, both *PpGA20ox1* and *− 2* were expressed in shoots, but *PpGA20ox1* levels were higher than *PpGA20ox2*. For the five *GA3ox* genes, only *PpGA3ox1* was highly expressed in shoots. Among these biosynthesis genes, *PpGA20ox1* and *PpGA3ox1* showed a gradual decrease in transcript level along shoots from top to bottom, and a similar trend was observed in bioactive GA_1_ and GA_4_ distribution. Among the GA-deactivation genes, *PpGA2ox6* was highly expressed in peach shoots*. PpGA2ox1* and *− 5* transcripts were relatively lower and showed a similar pattern to *PpGA20ox1* and *PpGA3ox1* in peach shoots*.* Overexpression of *PpGA20ox1, − 2,* or *PpGA2ox6* in Arabidopsis or tobacco promoted or depressed the plant growth, respectively, while *PpGA3ox1* did not affect plant height. Transient expression of *PpGA20ox1* in peach leaves significantly increased bioactive GA_1_ content.

**Conclusions:**

Our results suggest that *PpGA20ox* and *PpGA2ox* expression are closely associated with the distribution of active GA_1_ and GA_4_ in peach annual shoots. Our research lays a foundation for future studies into ways to effectively repress the rapid growth of peach shoot.

**Supplementary Information:**

The online version contains supplementary material available at 10.1186/s12864-022-08943-5.

## Background

Peach [*Prunus persica* (L.) Batsch.] is one of the most economically important fruit tree species. Its annual shoots grow rapidly in spring and summer, which causes many problems in peach production. This annual growth can close the tree canopy, which restricts the ventilation and light of the orchard, and requires increased labor for pruning trees. Paclobutrazol (PBZ), a biosynthetic inhibitor of GA, is usually used to slow peach stem growth. The primary function of GA in higher plants can be generalized as stimulating growth through the enhancement of cell elongation and cell division [1]. Therefore, it is likely that GA is an important hormone regulating the rapid growth of peach annual shoot.

Bioactive GAs are diterpene plant hormones. Their biosynthesis starts from geranylgeranyl diphosphate (GGDP), which is converted to *ent*-kaurene by two terpene synthases, *ent*-copalyl diphosphate synthase (CPS) and *ent*-kaurene synthase (KS), and then transformed into GA_12_ by two cytochrome P450 monooxygenases, *ent*-kaurene oxidase (KO) and *ent*-kaurenoic acid oxidase (KAO). Finally, GA_12_ is transformed into active GA_4_ by two 2-oxoglutarate-dependent dioxygenases, GA 20-oxidase (GA20ox) and GA 3-oxidase (GA3ox). GA_12_ also could be transformed into GA_53_ firstly by GA 13-oxidase (GA13ox) and then transformed into GA_1_ and GA_3_ by GA20ox and GA3ox [1, 2]. In addition, GA deactivation is an important mechanism for controlling endogenous GA content. The 2β-hydroxylation of active GA, a process catalyzed by GA 2-oxidase (GA2ox), is a primary way to inactivate GAs [3]. The deactivating function of CYP714 on active GAs has been demonstrated in rice and Arabidopsis [4–6]. An in vitro enzyme activity assay showed that EUI (CYP714D1) significantly reduced the biological activity of GA_4_ [4]. Mutation in GA biosynthetic/deactivation genes affects the endogenous GA content and therefore plant stature. A single nucleotide substitution in exon 5 of *OsKO2* leads to replacement of a highly conserved arginine with a serine, resulting in a dwarf phenotype [7]. The Arabidopsis *ga20ox1* mutant contains lower bioactive GA and shows a semi-dwarf phenotype [8, 9]. Four independent mutations in *ZmGA3ox2* give rise to a dwarf phenotype of maize [10]. *OsGA3ox2* is located at the *D18* locus, which is associated with a dwarf phenotype in rice due to a frameshift mutation [11]. *Rht18* semi-dwarfism in wheat is caused by increased *GA2oxA9* transcript levels and decreased endogenous GA contents [12].

GA transport is another way that plants modulate local bioactive GA levels. GA transport, in both acro- and basipetal directions, has been demonstrated to be essential for several developmental processes [13]. GAs are mobile signals from shoot to hypocotyl and can trigger local xylem expansion [14]. The acropetal translocation of GAs from wild-type rootstock to scions lacking a GA biosynthetic enzyme rescues the phenotypes [15].

Apart from the content of bioactive GAs, the activity and stability of each active GA form are important factors affecting GA signaling. More than 130 GA structures have been identified, with the most common active forms being GA_1_, GA_3_, and GA_4_ [1]. GA_1_ and GA_4_ are deactivated by GA2ox, while GA_3_, which is synthesized by GA3ox from GA_20_ via the intermediate GA_5_, is not deactivated by GA2ox [2]. Therefore, GA_3_ is more stable than GA_1_ and GA_4_. However, the activity of these three GA active forms differs. The universal occurrence of GA_1_ and GA_4_ in plants suggests that these are the functionally active forms. Additionally, GA_4_ appears to be more active than GA_1_ in rice [6].

GA is the primary growth-promoting hormone of peach annual shoots, but the genes involved in biosynthesis and degradation are poorly understood. To properly regulate the growth of annual shoots, it is necessary to identify the genes that encode the enzymes that synthesize and deactivate GA. Our previous study identified seven *PpGA2ox* genes in peach [16]. The present study identifies the GA biosynthetic genes and analyzes their transcription levels as well as those of the deactivating *PpGA2oxs* in six internodes from the top to the bottom of peach shoots. In addition, the contents of the GAs in different internodes were analyzed. Additionally, *PpGA20ox1*, *PpGA20ox2*, *PpGA3ox1,* and *PpGA2ox6* were overexpressed in Arabidopsis or tobacco. Finally, *PpGA20ox1* was transiently expressed in peach leaves, and the bioactive GAs were measured.

## Results

### Identification of GA biosynthetic genes in the peach genome

**Fig. 1:**
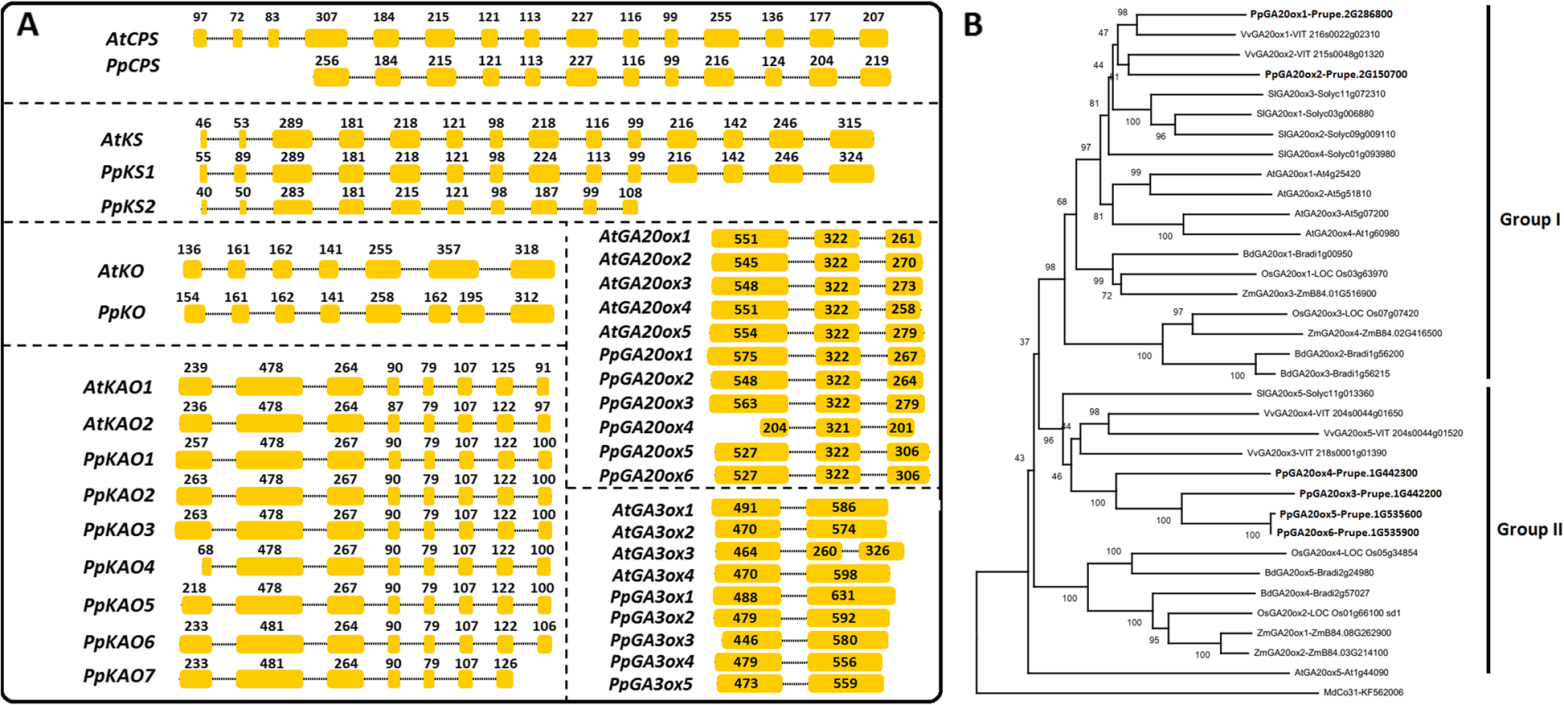
Identification of GA biosynthetic genes in peach. **A** Comparison of gene structure between Arabidopsis and peach. The yellow boxes represent exons and the dotted lines introns. The number in/above the yellow box represents the exon length (bp). **B** A phylogenetic tree of GA20ox. The amino acid sequences of GA20ox from seven species were downloaded from the Phytozome database. The Gene ID is listed after each gene name. GA20oxs are divided into two groups (I and II)

Six GA biosynthesis-related gene families (*CPS*, *KS*, *KO*, *KAO*, *GA20ox*, and *GA3ox*) were analyzed in peach. We identified one *CPS*, two *KS*s, one *KO*, seven *KAO*s, six *GA20ox*s, and five *GA3ox*s in the peach genome (Table 1; Fig**.** 1A). The number and length of the exons in each gene were predicted. There were 12 exons in *PpCPS*, which is three fewer than in Arabidopsis. *PpKS1* had the same number of exons as *AtKS*, while *PpKS2* had four fewer than *AtKS*. Among the seven *KAO* genes, *PpKAO1* to ***−*** *6* contained eight exons and had the same numbers as *AtKAO1* and ***−*** *2*. All eleven *GA20ox* genes in peach and Arabidopsis had three exons. Five *PpGA3ox* had two exons, the same as *AtGA3ox1*, ***−*** *2*, and ***−*** *4*. The GA biosynthetic genes showed high conservation of exon length, with any changes in exon length occurring mainly in the first and last exons.

**Table 1:** Six GA biosynthetic gene families in peach and Arabidopsis

No.	Gene name	Gene ID	CDS Length (bp)	No. of exon
1	*AtCPS*	At4g02780	2409	15
	*PpCPS*	Prupe.8G239900	2094	12
2	*AtKS*	At1g79460	2358	14
	*PpKS1*	Prupe.4G128500	2415	14
	*PpKS2*	Prupe.4G128600	1383	10
3	*AtKO*	At5g25900	1530	7
	*PpKO*	Prupe.1G388500	1545	8
4	*AtKAO1*	At1g05160	1473	8
	*AtKAO2*	At2g32440	1470	8
	*PpKAO1*	Prupe.2G109600	1506	8
	*PpKAO2*	Prupe.2G109700	1500	8
	*PpKAO3*	Prupe.2G108400	1506	8
	*PpKAO4*	Prupe.2G108600	1311	8
	*PpKAO5*	Prupe.2G108700	1461	8
	*PpKAO6*	Prupe.5G041400	1471	8
	*PpKAO7*	Prupe.5G041500	1383	7
5	*AtGA20ox1*	At4g25420	1134	3
	*AtGA20ox2*	At5g51810	1137	3
	*AtGA20ox3*	At5g07200	1143	3
	*AtGA20ox4*	At1g60980	1131	3
	*AtGA20ox5*	At1g44090	1158	3
	*PpGA20ox1*	Prupe.2G286800	1164	3
	*PpGA20ox2*	Prupe.2G150700	1134	3
	*PpGA20ox3*	Prupe.1G442200	1164	3
	*PpGA20ox4*	Prupe.1G442300	726	3
	*PpGA20ox5*	Prupe.1G535600	1155	3
	*PpGA20ox6*	Prupe.1G535900	1155	3
6	*AtGA3ox1*	At1g15550	1077	2
	*AtGA3ox2*	At1g80340	1044	2
	*AtGA3ox3*	At4g21690	1050	3
	*AtGA3ox4*	At1g80330	1068	2
	*PpGA3ox1*	Prupe.3G075600	1116	2
	*PpGA3ox2*	Prupe.2G061700	1068	2
	*PpGA3ox3*	Prupe.7G235400	1023	2
	*PpGA3ox4*	Prupe.1G467600	1032	2
	*PpGA3ox5*	Prupe.1G467800	1029	2

Interestingly, *PpKO* had 8 exons in peach (Fig**.** S1) and 7 exons in Arabidopsis. RT-PCR was used to amplify the coding sequence of *PpKO* and its gene structure was confirmed. The sixth exon in *AtKO* was 357 bp in length, which is equal to the sum of the sixth and seventh exons of *PpKO*. In the *GA3ox* family, only *AtGA3ox3* contains three exons, one more exon than the other *GA3ox* members. Alignment of the PpGA20ox with the AtGA20ox protein sequences identified two conserved motifs (NYYPXCQKP and LPWKET) postulated to be involved in binding the 2-oxoglutarate and the GA substrate [17]. The protein encoded by *PpGA20ox4* lacks the LPWKET motif (Fig**.** S2), implying that the function of *PpGA20ox4* may be changed.

Phylogenetic trees were constructed to analyze the evolutionary relationship of the six GA biosynthesis gene families. A phylogenetic tree was constructed using the amino acid sequence of just the GA20oxs from peach, grapevine, tomato, Arabidopsis, rice, maize, and the moss *Brachypodium distachyon* (Fig**.** 1B). All of the GA20ox protein sequences fell into one of two groups (group I and group II). Both groups contained GA20oxs from both Eudicots and Monocots, and seven analyzed species contained members from the two groups (Table 2). These results suggest that the GA20ox family evolved from two different ancestral *GA20ox* genes. PpGA20ox1 and PpGA20ox2 were clustered into group I, which also contained OsGA20ox1 and AtGA20ox1. PpGA20ox3, **−** 4, **−** 5, and **−** 6 were clustered into group II, which contained OsGA20ox2, the *SEMI-DWARF1* (*SD1*) gene in rice. Another phylogenetic tree of the CPS gene family showed that PpCPS clustered with CPS from apple and strawberry (Fig**.** S3A). Peach *KS* and *KO* genes clustered together with KS and KO of apple and strawberry (Fig**.** S3B and C). Five PpKAOs (PpKAO1, **−** 2, **−** 3, **−** 4, and **−** 5) clustered with the KAOs from different plants, and the remaining 2 PpKAOs (PpKAO6 and **−** 7) were separated out (Fig**.** S3D). The GA3ox phylogenetic tree divided all GA3oxs into two groups, which contained the GA3oxs from Eudicots and Monocots, respectively (Fig**.** S3E).

**Table 2:** Distribution of GA20ox between the two phylogenetic groups

Species	Group I	Group II
Arabidopsis	AtGA20ox1, −2, −3, −4	AtGA20ox5
Peach	PpGA20ox1,-2	PpGA20ox3, −4, −5, −6
Grape	VvGA20ox1, −2	VvGA20ox3, −4, −5
Tomato	SlGA20ox1, −2, −3, −4	SlGA20ox5
*B. distachyon*	BdGA20ox1, − 2, − 3	BdGA20ox4, −5
Rice	OsGA20ox1, − 3	OsGA20ox2, − 4
Maize	ZmGA20ox3, − 4	ZmGA20ox1, − 2

### Transcription profile of GA biosynthetic genes in different tissues

**Fig. 2:**
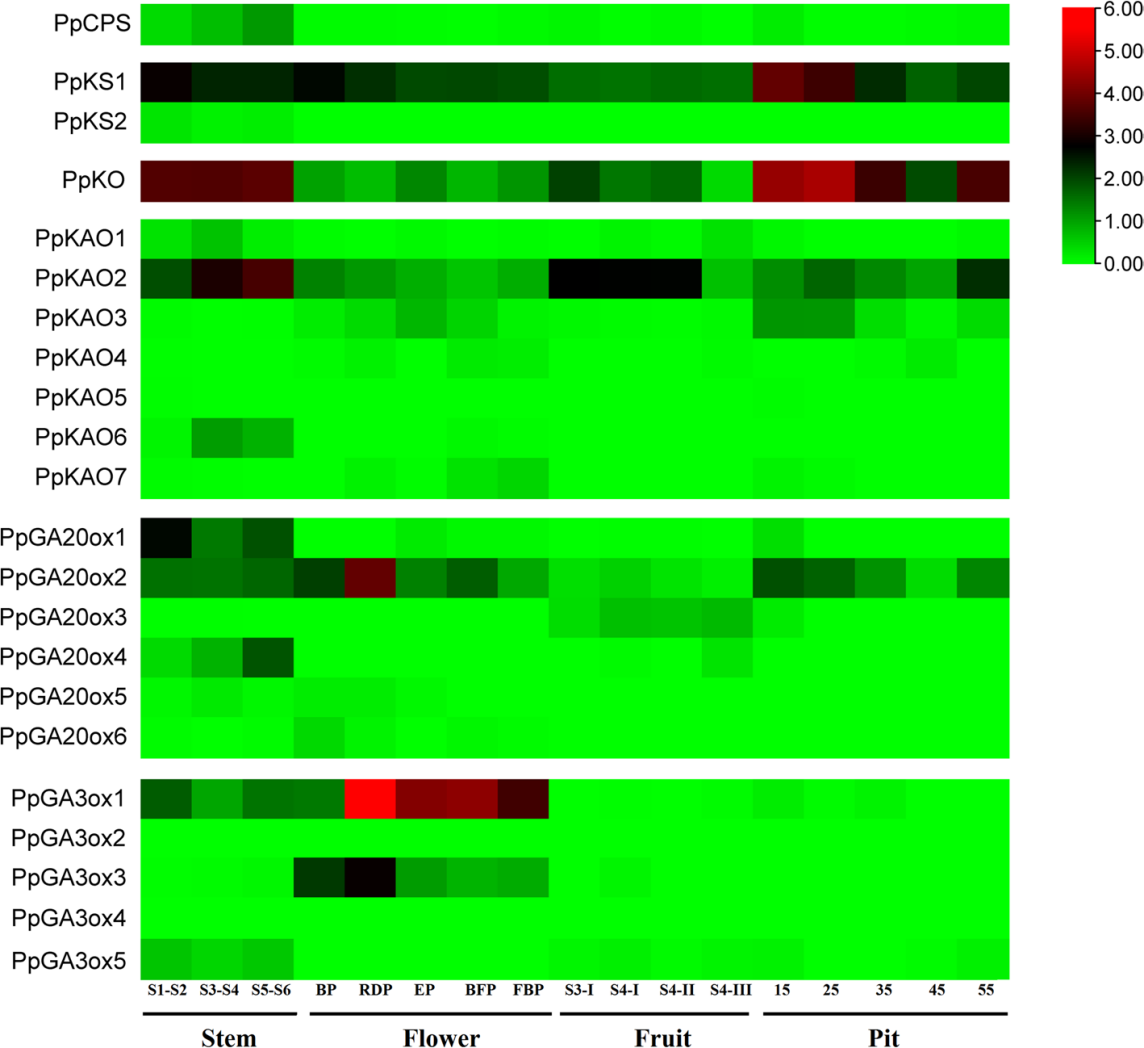
Heat map of the expression levels of *CPS*, *KS*, *KO*, *KAO*, *GA20ox* and *GA3ox* genes in stem, flower, fruit and fruit pit. Red and green shading represent high and low expression levels, respectively. S1-S2, S3-S4 and S5-S6 represent combined samples of the internodes S1 and S2, S3 and S4, and S5 and S6. BP = bud period; RDP = red dot period; EP = equivalent in size between petal and sepal period; BFP = budding flower period; FBP = full bloom period. S3-I, S4-I, S4-II, and S4-III represent 118, 120, 122 and 124 d after full bloom, respectively. Pits were collected at 15, 25, 35, 45, and 55 days after full bloom

Because *KS*, *KAO*, *GA20ox,* and *GA3ox* gene families are comprised of multiple members, the transcription profiles of these genes were analyzed using the transcriptomes derived from stem, flowers, fruit, and fruit pits (Fig**.** 2). All genes showed at least some expression. Among the seven *KAO*s, *PpKAO2* was highly expressed in all analyzed samples, while *PpKAO3* was mainly expressed in flower and fruit pits. Among the five *GA3oxs*, *PpGA3ox1* was mainly expressed in flowers and stems, and *PpGA3ox3* was expressed in flowers. Between the two *PpKS*s, *PpKS1* was expressed in all analyzed tissues. Among the six *PpGA20ox*s, *PpGA20ox2* was expressed in all analyzed samples, while *PpGA20ox1* was mainly expressed in stems. *PpCPS* was expressed in all analyzed tissues, while *PpKO* was highly expressed in stem and fruit pit.

### Determination of active GA levels in annual peach shoot

**Fig. 3:**
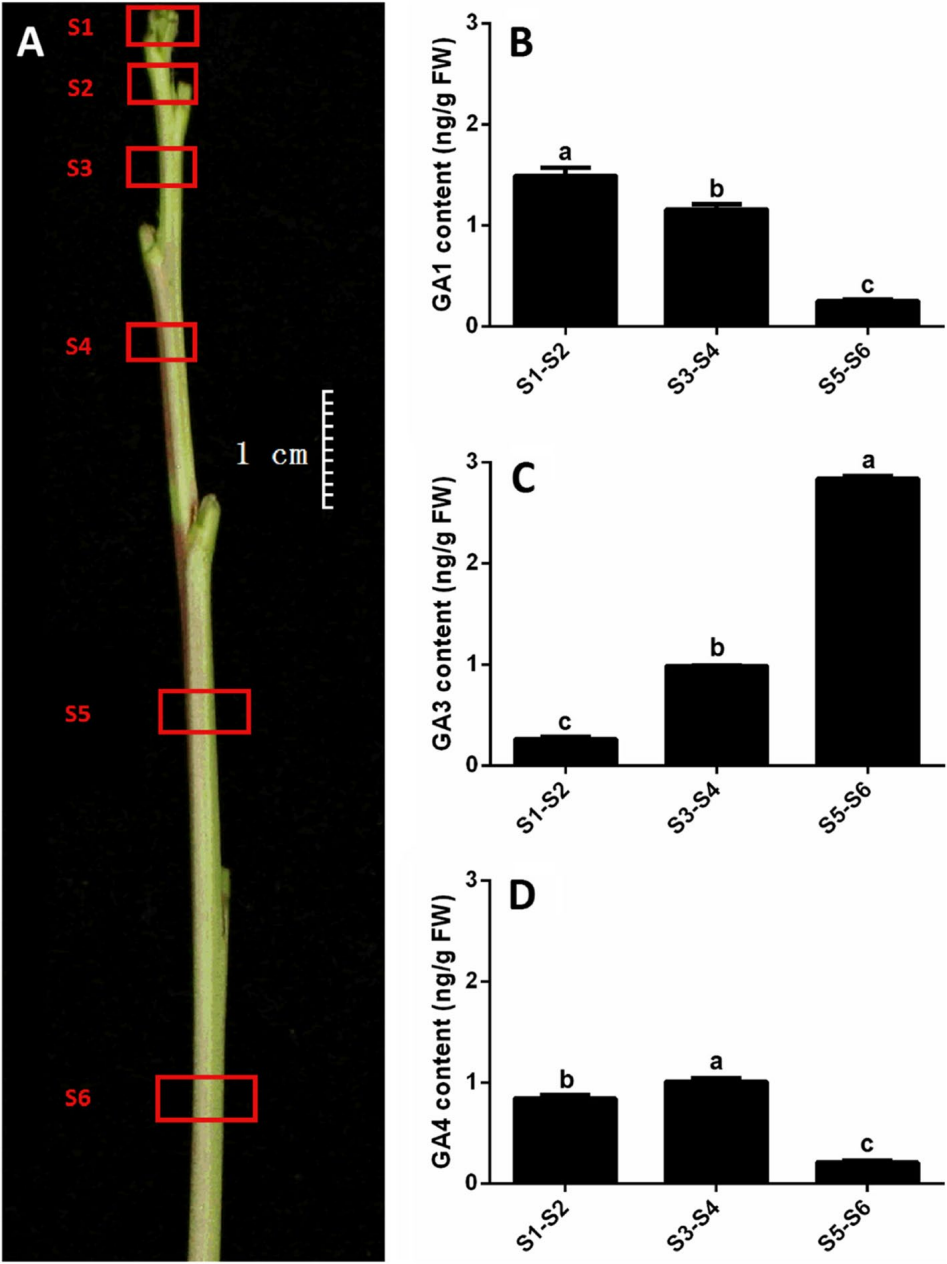
The distribution of GA_1_, GA_3_ and GA_4_ in annual peach shoots. **A** A sylleptic shoot, with red boxes indicating the six sampling locations. The GA_1_ (**B**), GA_3_ (**C**) and GA_4_ (**D**) content in S1-S2, S3-S4 and S5-S6. S1-S2, S3-S4 and S5-S6 represent combined internode samples. Different lowercase letters indicate significant differences according to Fisher’s LSD test (*P* < 0.05)

Annual shoots of peach grow quickly in the spring and summer, implying active synthesis of GAs in the shoots. Sylleptic shoots undergoing vigorous growth were used to determine the content of active GAs. Segments (0.2 cm in length) were collected from the center of six internodes from the tip to the lower internodes of the branch and were successively named S1, S2, S3, S4, S5, and S6 (Fig**.** 3A). Due to their low concentrations, every two internodes were combined to determine the GA content. The GA_1_ content gradually decreased from apex to bottom, with a level 6 times higher in tip internodes (1.5 ng/g FW) than in the lowest internodes (0.25 ng/g FW) (Fig**.** 3B). GA_4_ is highest in the middle (1.01 ng/g FW) and lowest in the bottom internodes (0.21 ng/g FW) (Fig**.** 3D). These results demonstrated that GA_1_ and GA_4_ are at high levels in the final internodes of annual shoots.

Interestingly, the GA_3_ content gradually increased from the tip to the branch bottom (Fig**.** 3C). The GA_3_ level in the top internodes was significantly lower than were the levels of GA_1_ and GA_4_. The GA_3_ level in the bottom internodes (2.84 ng/g FW) was 11.36 times higher than in the top internodes (0.25 ng/g FW). These results demonstrated that the lower internodes accumulated a large amount of active GA_3_.

### Transcription level of GA biosynthetic and deactivation genes in six internodes along the elongating shoot from top to bottom

**Fig. 4:**
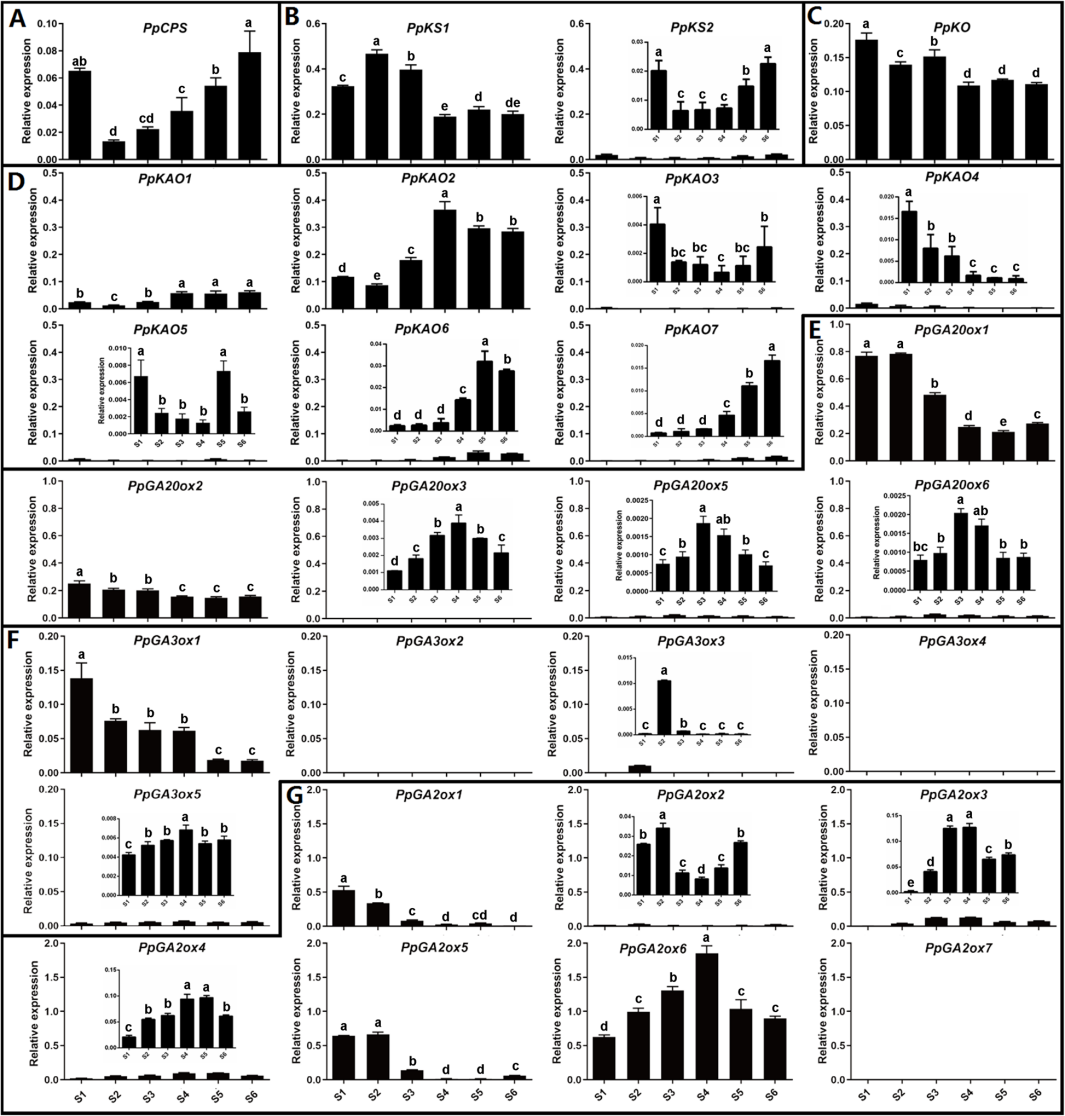
The transcription pattern of GA metabolic genes in six internodes of annual peach shoots from apex to bottom. GA biosynthesis gene families include *CPS* (A), *KS* (B), *KO* (C), *KAO* (D), *GA20ox* (E) and *GA3ox* (F). *GA2ox* (G) is involved in GA deactivation. Different lowercase letters indicate significant differences according to Fisher’s LSD test (*P* < 0.05)

The transcript levels of the GA biosynthetic genes were analyzed in six internodes along the elongating shoot from top to bottom (Fig**.** 4). *PpCPS* showed a gradually increasing trend from S2 to S6, and was at its highest level in S6. *PpKS1* showed a clearly higher level in all six internodes than *PpKS2* and had the highest level in S2. *PpKO* was expressed in all six internodes. *PpKAO1* and ***−*** *2* were expressed in all six internodes, with *PpKAO2* higher in all analyzed internodes. Expression of *PpKAO3*, ***−*** *4*, ***−*** *5*, ***−*** *6,* and ***−*** *7* was relatively low in all six internodes.

Among the six *GA20ox* genes, *PpGA20ox1* was highly expressed in the tip internodes (S1 and S2) and weakly in lowest internodes. *PpGA20ox2* was expressed in all analyzed internodes. *PpGA20ox5* and *PpGA20ox6* are tandem repeat genes, with a coding sequence similarity of 99% and similar transcription profiles in the stem. The level of *PpGA20ox3* was very low in all six internodes. Among the five *GA3ox* genes, *PpGA3ox1* shows a gradually decreasing trend in transcript level from top to bottom, and *PpGA3ox5* is present at a very low level in all six internodes.

Our previous study identified seven *PpGA2ox* genes in peach [16]. Among the seven *GA2ox* genes, *PpGA2ox1* and *PpGA2ox5* showed a gradually decreasing trend in transcript level from top to bottom. *PpGA2ox6* had the highest expression level in all analyzed internodes compared with the other six *GA2ox* genes. Some *PpGA2oxs* genes showed very little transcription.

### Overexpression of *PpGA20ox1*, − *2*, *− 5*, *PpGA3ox*1, and *PpGA2ox6* in Arabidopsis or tobacco

**Fig. 5:**
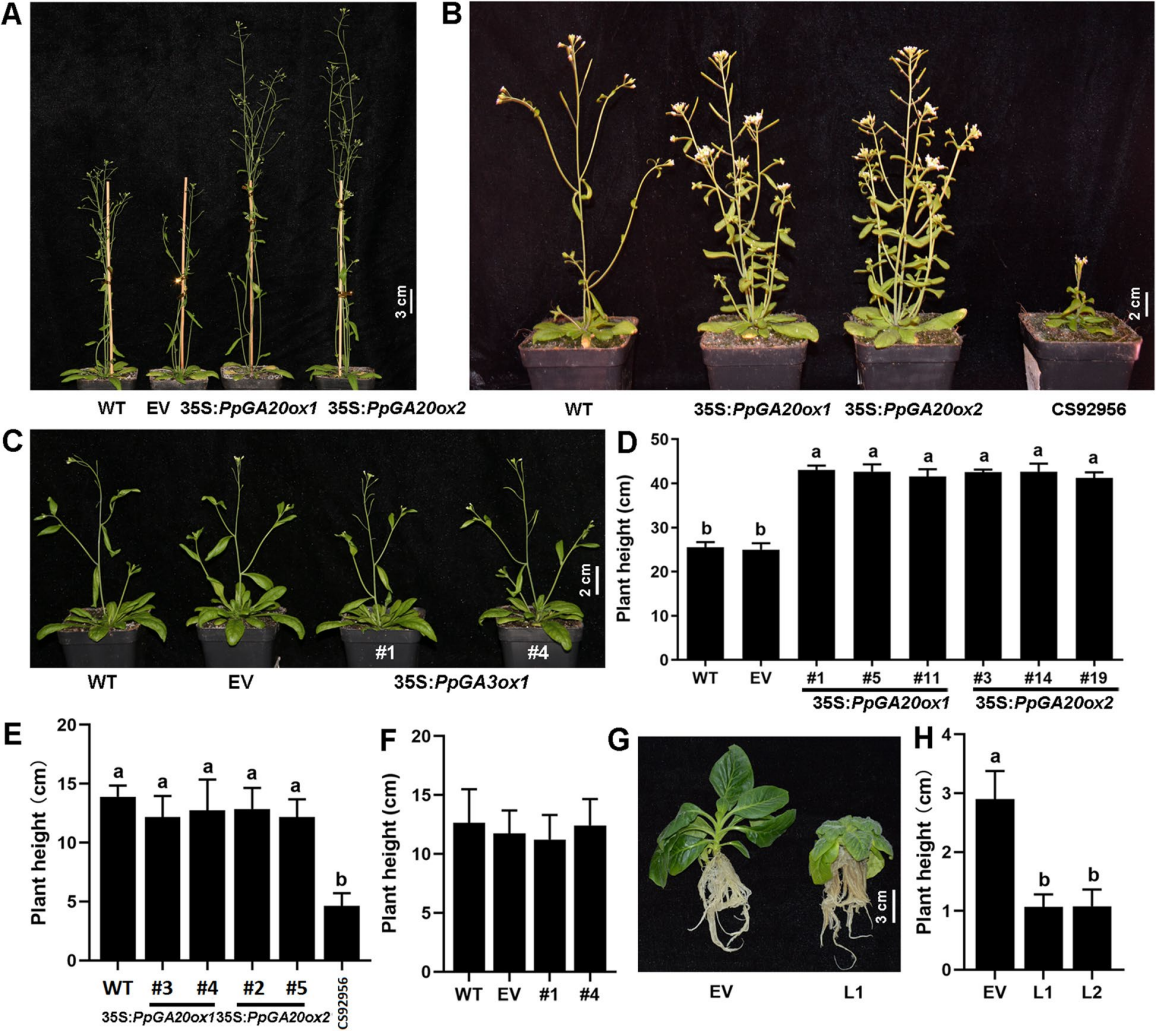
Overexpression of *PpGA20ox1*, *PpGA20ox2*, *PpGA3ox1* and *PpGA2ox6* in Arabidopsis or tobacco. Photograph (**A**) and height (**D**) of 50-day-old seedlings of WT, empty vector (EV) control, and lines overexpressing *PpGA20ox1* and *PpGA20ox2* in wild-type background, from left to right). Photograph (**B**) and height (**E**) of 42-day-old seedlings of WT, overexpression of *PpGA20ox1* and *PpGA20ox2* in *ga20ox* mutant background, and *ga20ox* mutant, from left to right. Photograph (**C**) and height (F) of 40-day-old seedlings of WT, EV control, and two lines overexpressing *PpGA3ox1* in wild-type background, from left to right). Photograph (**G**) and height (**H**) of tobacco overexpressing *PpGA2ox6*. Different lowercase letters indicate significant differences according to Fisher’s LSD test (*P* < 0.05)

To determine if the putative *PpGA20oxs* encode functional enzymes, 35S-*PpGA20ox1* and ***−*** *2* were transformed into wild-type Arabidopsis (WT) (Fig**.** 5). Empty vector (EV) served as control. Three transgenic lines each of *PpGA20ox1* and ***−*** *2* were obtained (Fig**.** S4C and D). The T3 seedlings were grown for 50 d, then the length of the main stem was measured. The stems of all transgenic lines ranged from 36.6**–**46 cm in length, which is significantly longer than that of WT and control (Fig**.** 5A, D). *PpGA20ox1* and ***−*** *2* were also transformed into an Arabidopsis *ga20ox* mutant (*CS92956*) (Fig**.** S4A and B; Fig**.** 5B, E) and the phenotype and plant height of 42-day-old seedlings were analyzed. Overexpression of *GA20ox1* and ***−*** *2* recovered the length of the main stems to that of wild type. Based on the phylogenetic tree of GA20ox, *PpGA20ox1* and ***−*** *2* belong to group I, and *PpGA20ox3*, ***−*** *4*, ***−*** *5,* and ***−*** *6* are clustered into groups II. 35S-*PpGA20ox5* was transformed into wild type, and the phenotype and plant height of 28-day-old seedlings were analyzed. The transgenic lines had the same phenotype as wild type overexpressing *PpGA20ox1* and ***−*** *2* (Fig**.** S5).

*PpGA3ox1* was transformed into Arabidopsis (Fig**.** 5C, F). Three *PpGA3ox1* transgenic lines were obtained (Fig**.** S4E). The lengths of the main stem at 40 d were not significantly different between any of the transgenic lines and WT or EV. *PpGA2ox-6* was transformed into tobacco (*Nicotiana tabacum*) (Fig**.** S4; Fig**.** 5G, H). Two transgenic lines were selected and showed a dwarf phenotype compared with the control.

### Transient expression of *PpGA20ox1* in peach leaves

**Fig. 6:**
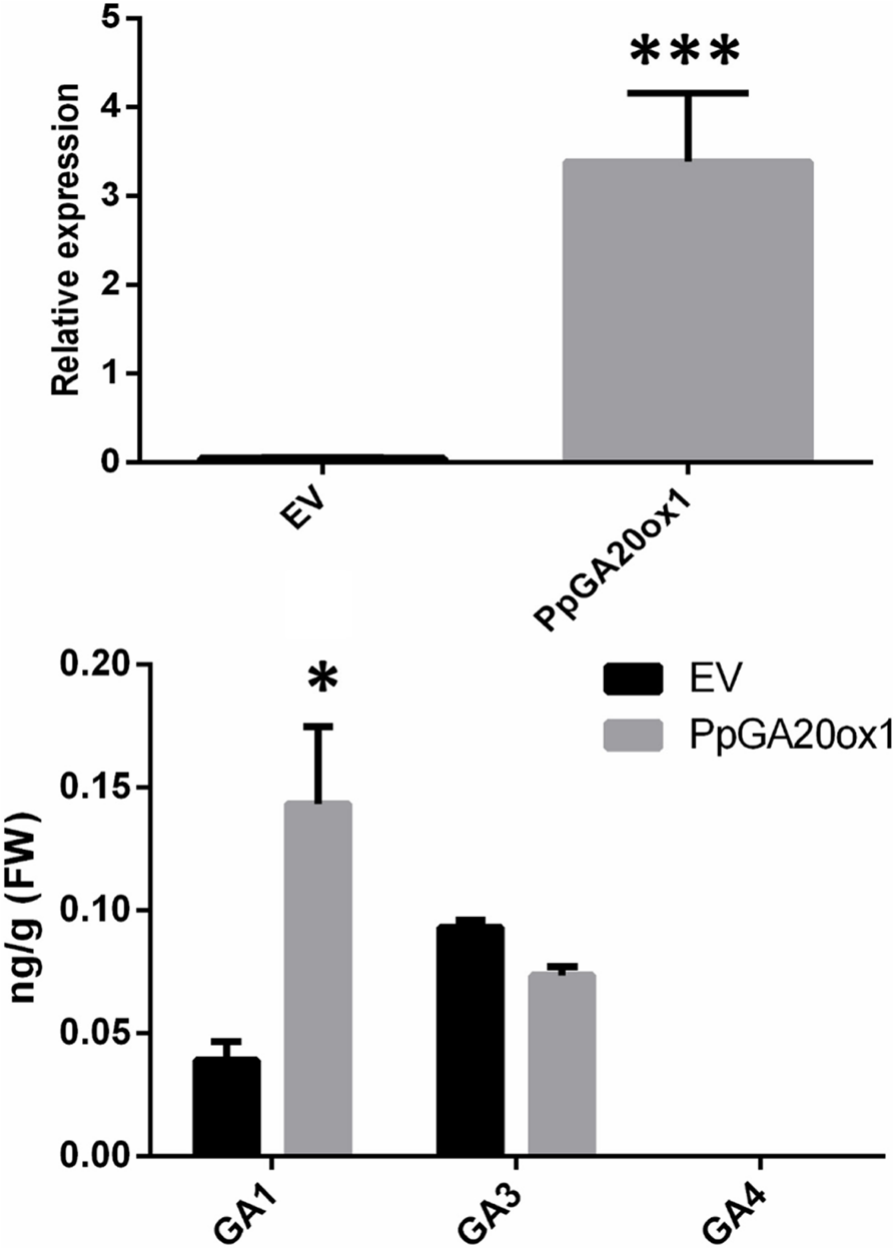
Bioactive GA contents of peach leaves transiently expressing *PpGA20ox1*. **A** The transcription level of *PpGA20ox1* in peach leaves (*n* = 3). **B** Active GA content in peach leaves. * and *** indicate a significant difference at *P* < 0.05 and *P* < 0.001 between EV and *PpGA20ox1* as determined by Student’s t-test

The effect of *PpGA20ox1* on bioactive GA contents was confirmed in peach by a transient expression assay. 35S-*PpGA20ox1* and EV were transiently expressed in peach leaves. The contents of three active GAs were analyzed (Fig**.** 6). The result showed that there was a significant difference (*P* < 0.05) in the content of GA_1_, which was 3.7 times higher in leaves overexpressing *PpGA20ox1* than in the control. GA_3_ was slightly lower in leaves overexpressing *PpGA20ox1* compared to the control, but didn’t differ significantly between 35S-*PpGA20ox1* and EV. The content of GA_4_ was too low to detect. Our results demonstrated that overexpressing *PpGA20ox1* increases the GA_1_ content in peach leaves.

## Discussion

The rapid growth of annual shoots in peach is detrimental to agricultural production. Finding ways to repress this rapid growth is necessary. As GAs play important roles in the rapid growth process, it is important to study the genes involved in GA biosynthesis and degradation. In this study, GA biosynthetic gene families were firstly determined. To find out the candidate members involved in GA metabolism in peach shoots, the expression patterns of the GA metabolic genes were determined along a growing stem. Then, these found out candidate members were transformed into Arabidopsis or tobacco to determine gene function.

### The *PpGA20ox1* transcript pattern reflects active GA_1_ and GA_4_ distribution in annual peach shoots

Six gene families (*CPS*, *KS*, *KO*, *KAO*, *GA20ox* and *GA3ox*) are involved in GA biosynthesis. Among these genes, only *PpGA20ox1* and *PpGA3ox1* showed a gradual decrease in transcription level from shoot tip to base, a gradient that is similar to the distribution of bioactive GA_1_ and GA_4_ in the annual shoots. The results suggested that *PpGA20ox1* and *PpGA3ox1* may be the key genes controlling GA contents.

The function of GA20ox has been extensively studied in many plants, and overexpression of *GA20ox* can increase endogenous GAs in Arabidopsis, potato, tomato, rice, *Artemisia annua*, tobacco, carrizo citrange, cotton, poplar, kenaf, and switchgrass [18–29]. However, overexpression of *AtGA3ox*, the last enzyme in the GA biosynthesis pathway, showed no significant change in active GAs or plant stature in hybrid aspen [30]. Our results also demonstrated that *PpGA20ox1* and ***−*** *2*, but not *PpGA3ox1*, promoted the growth of Arabidopsis. To demonstrate the function of *PpGA20ox* in peach, *PpGA20ox1* was transiently expressed in peach leaves. Overexpression of *PpGA20ox1* significantly increased GA_1_ content. Altogether, our results suggest that *PpGA20ox1* encodes a key enzyme for GA biosynthesis in annual peach shoot.

Intriguingly, *PpGA3ox1* had a similar expression pattern as *PpGA20ox1* in the annual peach shoots. These similar *GA20ox* and *GA3ox* expression patterns are reported in several studies. In Arabidopsis, DELLA-dependent regulation also promotes the simultaneous transcription of *GA20ox* and *GA3ox* [31–34]. Treatment with low ratio of red: far red light upregulated the transcript levels of *GA20ox* and *GA3ox* simultaneously in *Rumex palustris* [35]. Inversely, the transcript levels of *GA20ox* and *GA3ox* are downregulated due to mutation of the DELLA proteins LA and CRY in pea [36]. Our previous study showed that the transcript levels of *PpGA20ox1* and *PpGA3ox1* are significantly upregulated in the dwarf peach ‘FenHuaShouXingTao’ that carries a mutation in the GA receptor PpGID1c [31]. All these results suggest that GA3ox is also important for GA homeostasis.

### GA_3_ content is relatively higher in the sixth internode of peach shoots

GA_3_ was reported to highly accumulate in the lower internodes of poplar branches [37]. GA transport is well-demonstrated, and many forms of GA are deemed as mobile entities. In general, precursors are suggested to be the mobile forms [38]. GA_20_ acts as the major mobile form in pea [15]. GA_12_ is the major form transported over a long distance from roots to shoots in Arabidopsis [39]; in cucumber, GA_9_ is produced in the ovaries and moves to the sepals and the petals, whereas GA_9_ is converted into the bioactive GA_4_ to regulate organ growth [40]. GA_3_, an active GAs, is synthesized by GA3ox from GA_20_ via GA_5_ [2]. It is well known that GA_1_ and GA_4_, but not GA_3_, can be converted by GA2ox into the inactive GAs, namely GA_8_ and GA_34_ [1]. Our previous study demonstrated that three peach GA2oxs could inactivate GA_1_, but not GA_3_ [16]. In this study, GA_3_ content gradually increased from the tip to the branch bottom. *PpGA2ox6* was highly expressed in all analyzed internodes of peach shoot. In addition, GA_5_, an intermediate product from GA_20_ to GA_3_, has been shown to resist deactivation by GA2ox and could be transported into the meristem [41]. Together, the high accumulation of GA_3_ in the lower internodes may be partly attributed to GA_3_ transportation in the annual shoots of peach.

## Conclusions

In this study, peach GA biosynthetic genes were analyzed in detail. The upstream biosynthetic genes have only one or two copies (1 *CPS*, 2 *KS*s and 1 *KO*), while the downstream genes have multiple copies (7 *KAO*s, 6 *GA20ox*s and 5 *GA3ox*s). Among the six biosynthetic genes, only *PpGA20ox1* and *PpGA3ox1* show a gradual decrease in the transcript level from apex to bottom of annual shoots of peach, which was synchronized with the GA_1_ and GA_4_ distribution. Among the GA metabolic genes, *PpGA2ox1* and *PpGA2ox5* showed similar transcript patterns to *PpGA20ox1* and *PpGA3ox1.* Overexpression of *PpGA20ox1, − 2* or *PpGA2ox6* in Arabidopsis or tobacco promoted or repressed plant growth, respectively, while *PpGA3ox1* had no effect on the plant growth. Transient expression of *PpGA20ox1* in peach leaves significantly increased the content of GA_1_. Our study demonstrates that *PpGA20ox* and *PpGA2ox* encode key enzymes that are associated with active GA distribution in annual shoots. This information may prove helpful for devising ways to regulate the endogenous GA levels, and thus annual growth, in peach shoots.

## Methods

### Plants material

Peach trees of the cultivars ‘QiuMiHong’ (QMH) and ‘YuJinMi3’ (YJM3) are maintained in the Fruit Tree Germplasm Repository of Henan Agricultural University (Henan Province, China). The stem and fruit samples were collected from ‘QMH’ and fruit pits were collected from ‘YuJinMi3’. Fruit developmental stages were divided according to Gabotti et al. [42]. From the S3 to S4 stage, fruit mesocarp tissues were collected at 118 d (S3-I), 120 d (S4-I), 122 d (S4-II), and 124 d (S4-III) after full bloom. Five fruit pit samples at 15, 25, 35, 45, and 55 d after the full bloom period were collected. From the top to bottom of stems showing rapid growth, six internodes were collected and divided into three parts [top (S1-S2), middle (S3-S4), and bottom (S5-S6)]. The samples of stems and fruit pits were prepared between April and June 2019, while fruit samples were prepared on April 28, 2020. All samples were used for transcriptome analysis and the stem samples were also used for GA content analysis.

Tobacco [*Nicotiana tabacum*] ‘K326’ and peach seedlings were cultured in a growth chamber at 24 °C under a photoperiod of 16 h/8 h. Arabidopsis seedlings were cultured in greenhouse at 23 °C, 16 h of light/8 h of dark, relative humidity of 55%.

### Sequence retrieval

The peach genome sequence was downloaded from the GDR database (https://www.rosaceae.org). A standalone BLAST software was used to conduct local blast searches using the coding sequence of *CPS* (At4g02780), *KS* (At1g79460), *KO* (At5g25900), *KAO* (At1g05160), *GA20ox* (AT4G25420) and *GA3ox* (At1g15550) as the query sequences against the peach genome (Table S1). For *CPS*, *KS*, and *KO*, genes with the highest sequence similarity to the homologous genes in Arabidopsis were assigned as significant. *PpKS2*, a tandem duplicated gene, was also mentioned in this study. For *KAO*, scores higher than 545 with an “E” value of 0 were assigned as significant. For *GA20ox* and *GA3ox*, scores higher than 281 with an “E” value of e-93 were assigned as significant. The gene structure was confirmed using the sequence alignment/map (SAM) files of the transcriptomes and using RT-PCR to amplify the coding sequence. Finally, the Pfam (http://pfam.sanger.ac.uk/search) and SMART (http://smart.emblheidelberg.de/) databases were used to confirm the conserved domains.

### Construction of phylogenetic tree

Phylogenetic trees were constructed using the amino acid sequences of CPS, KS, KO, KAO, GA20ox and GA3ox from different plants. The sequences were downloaded from the NCBI database (https://www.ncbi.nlm.nih.gov/) and the Phytozome database (https://phytozome-next.jgi.doe.gov/). Sequence alignment was performed using Clustal X. Neighbor-joining trees were constructed using the heuristic search strategies of MEGA version 5. Bootstrap values were calculated from 1000 replicate analyses. For the phylogenetic tree of GA20ox, a 2OG-Fe (II) oxygenase gene of apple (GenBank accession no. KF562006) served as an outgroup.

### RNA extraction and RNA seq

Total RNA was extracted using the RNA extraction kit (Zoman, Beijing, China). The integrity of each RNA was assessed by electrophoresis on a 1.2% agarose gel, and RNA quality was evaluated in a NanoDrop 2000c spectrophotometer (Thermo Scientific, Waltham, MA). RNA samples, with A_260_/_280_ and A_260_/_230_ ratios between 1.8**–**2.2, were selected for the construction of cDNA libraries and RNA-sequencing. mRNA was purified and then assessed using an Agilent Technologies 2100 Bioanalyzer (Agilent, United States) to generate a transcriptome. The constructed RNA library was sequenced using a BGI-SEQ-500 platform at Shenzhen Genomics Institute (BGI, Shenzhen, China) after a library quality test. Low-quality reads and adapters of the RNA-Seq raw data were removed first for all samples to obtain high-quality clean data when RNA-seq was completed. Clean data were compared to the reference genome by HISAT software, and then the R package HTSeq was used to calculate the fragments per kilobase of exon model per million mapped fragments (FPKM) for each transcript.

### Expression profiles of GA biosynthetic gene families

The transcriptional data of GA biosynthetic gene families, *CPS*, *KS*, *KO*, *KAO*, *GA20ox*, *GA3ox*, and *GA2ox*, were extracted from the transcriptome data of stems, fruits, flowers and fruit pits. Among these transcriptome dataset, flowers transcriptome data were sequenced by Lian et al. [43]. Flowers were collected from two peach cultivars, ‘CN14’ and ‘HuangShuiMi’ (HSM). ‘CN14’ is a semi-dwarf cultivar and ‘HSM’ is a standard cultivar. Dwarfing alleles in ‘CN14’ can cause major changes in GA biosynthetic gene expression levels, so the transcriptional data of GA biosynthetic gene families from ‘HSM’ are used in this study. Heat maps was drawn based on the log2^(FPKM)^ using pheatmap (*v* 1.0.12) in the R statistical language.

### Analysis of bioactive GA content

Approximately 1.5 g of the tissues were ground in liquid nitrogen. The ground powder was transferred into a 50-mL centrifuge tube and mixed with 10 mL of extraction solution (isopropanol/ hydrochloric acid mixture). The mixture was shaken at 4 °C for 30 min. Dichloromethane (20 mL) was added to the mixture, which was further shaken at 4 °C for 30 min. The mixture was centrifuged at 10000**×**g for 5 min at 4 °C and separated into two layers. The lower layer was dried using an N_2_ gas stream and redissolved in 400 μL methanol containing 0.1% formic acid. The extracts were filtered through 0.22 μm Millipore membranes and analyzed using an HPLC ESI-MS/MS system (Agilent, America) equipped with a Poroshell 120 SB-C18 column (2.1 × 150 mm, with a particle size of 2.7 μm; Agilent, America). The column was sequentially eluted using mobile phase A (methanol containing 0.1% formic acid) and mobile phase B (water containing 0.1% formic acid). The linear gradient for phase B was as follows: 0**–**2 min, 80%; 2**–**14 min, 80**–**20%; 14**–**15 min, 20%; 15.1 min, 80%; and 15.1**–**20 min, 80%. Mass spectra were acquired in positive ion mode. The mass spectrometer ion spray temperature was 400 °C, the ion spray voltage was 4500 V, and the curtain gas was 15 psi. Standard curves were constructed and used to quantify the content of GA_1_, GA_3_ and GA_4_. Deuterated GA_4_ (10 ng) was added into 1 mL of the extracts, and the parent/ daughter ions of deuterated GA_4_ were 335.1/245.2 and 335.1/213.1. Parent/Daughter ions for GA_1_ were 347.4/259.2 and 347.4/273.1, GA_3_ were 345.2/143.0 and 345.2/239.2, and GA_4_ were 331.4/243.2 and 331.4/213.1.

### Analysis of gene transcription level

Quantitative real-time RT-PCR (qRT-PCR) was carried out with the StepOnePlus Real-Time PCR System (Applied Biosystems, Foster, CA) as described previously [31] using an ABI PRISM 7500 FAST Sequence Detection System (Applied Biosystems, USA). The primer sequences are shown in Table S2. The peach actin gene *PpGAPDH* (Prupe.8G132000) was used to normalize the RNA-expression levels. Melt curve analysis was performed at the end of 40 cycles to ensure the proper amplification of target fragments. The melt curve had a single, narrow, sharp peak, indicating that the primers used for qRT-PCR were of good quality and met the requirements of qRT-PCR assay. Each experiment was repeated three times with three biological replicates.

### Expression vector construction and plant transformation

The whole coding sequences of *PpGA20ox1*, ***−*** *2*, ***−*** *5*, *PpGA3ox1* and *PpGA2ox6* were amplified using cDNA synthesized from shoot tips of ‘QMH’ as templates. The PCR product and pSAK277 vector were digested with a restriction enzyme, and then joined together using ligases (NEB, Beijing). Arabidopsis transformation was performed according to the floral dip method [44]. For transgenic plant selection, T0 seeds were sterilized and germinated on Murashige and Skoog (MS) medium containing 12 μg mL^**− 1**^ kanamycin. Kanamycin-resistant plants were transplanted to soil and placed in a growth chamber at 25 °C and 65**–**80% relative humidity. Phenotype analysis was conducted in the T3 generation.

Tobacco (*Nicotiana tabacum*) was transformed using the leaf-disc method [45]. More than five transgenic plants were selected using Murashige and Skoog (MS) medium containing 50 μg mL^**− 1**^ kanamycin and were confirmed by PCR using specific primers for the *PpGA2ox6* gene.

### Transient expression of *PpGA20ox1* in peach leaves

The vector *35S:PpGA20ox1* was introduced into the GV3101 *Agrobacterium* strain by heat shock transformation. Peach seedlings were cultivated for 18 d and then immersed into the bacterial suspension carrying either a control plasmid or recombinant plasmid at room temperature. A vacuum chamber attached to a vacuum pump (Model#SHZ-D, China) was used to create a vacuum (0.07 MPa) for 30 min to aid plant uptake of the suspension. Finally, the peach seedlings were cultured in a growth chamber at a temperature of 22 °C under a 16-h light/8-h dark cycle for 2 d. Leaves were collected for analysis of gene transcription levels and GA content.

## Supplementary Information

**Additional file 1: Figure S1.** Verification of *PpKO* gene structure using the alignment results of reads obtained from the transcriptome of peach flowers, stems, fruits and pits. **Figure S2.** Sequence alignment of GA20ox from Arabidopsis and peach. The LPWKET motif are underlined. **Figure S3.** The phylogenetic trees of CPS (A), KS (B), KO (C), KAO (D) and GA3ox (E). The amino acid sequences were downloaded from Phytozome database and the Gene ID are listed after the gene name. **Figure S4.** qPCR analysis of transgenic lines. Two transgenic lines of *PpGA20ox1* (A) and -2 (B) in *ga20ox* mutant (CS92956) of Arabidopsis. Three transgenic lines of *PpGA20ox1* (C) and -2 (D) in Arabidopsis (Ecotype Columbia). Two lines of *PpGA3ox1* (E) in Arabidopsis (Ecotype Columbia). Two lines of *PpGA2ox6* in *Nicotiana tabacum*. **Figure S5.** Overexpression of *PpGA20ox5* in Arabidopsis. **Table S1.** Sequence of genes involved in GA biosynthesis of peach. **Table S2.** List of primers used in this study.

## Data Availability

The RNA-seq data used in this study are available through BioProject accession number PRJNA723104 on NCBI SRA (Sequence Read Archive) and included in the following published article: Lian, X., Zhang, H., Jiang, C., Gao, F., Yan, L., Zheng, X., Cheng, J., Wang, W., Wang, X., Ye, X., Li, J., Zhang, L., Li, Z., Tan, B., Feng, J. (2021). De novo chromosome-level genome of a semi-dwarf cultivar of *Prunus persica* identifies the aquaporin *PpTIP2* as responsible for temperature-sensitive semi-dwarf trait and *PpB3–1* for flower type and size. Plant biotechnology journal, 10.1111/pbi.13767. Advance online publication. 10.1111/pbi.13767.
