# Supplementary material for: The distribution of bioactive gibberellins along peach annual shoots is closely associated with PpGA20ox and PpGA2ox expression profiles
Source: BMC Genomics. 2022 Oct 28;23:730. doi: 10.1186/s12864-022-08943-5 (PMC9615383; doi:10.1186/s12864-022-08943-5)
Supplement: Supplementary file 1 — Additional file 1: Figure S1. Verification of PpKO gene structure using the alignment results of reads obtained from the transcriptome of peach flowers, stems, fruits and pits. Figure S2. Sequence alignment of GA20ox from Arabidopsis and peach. The LPWKET motif are underlined. Figure S3. The phylogenetic trees of CPS (A), KS (B), KO (C), KAO (D) and GA3ox (E). The amino acid sequences were downloaded from Phytozome database and the Gene ID are listed after the gene name. Figure S4. qPCR analysis of transgenic lines. Two transgenic lines of PpGA20ox1 (A) and -2 (B) in ga20ox mutant (CS92956) of Arabidopsis. Three transgenic lines of PpGA20ox1 (C) and -2 (D) in Arabidopsis (Ecotype Columbia). Two lines of PpGA3ox1 (E) in Arabidopsis (Ecotype Columbia). Two lines of PpGA2ox6 in Nicotiana tabacum. Figure S5. Overexpression of PpGA20ox5 in Arabidopsis. Table S1. Sequence of genes involved in GA biosynthesis of peach. Table S2. List of primers used in this study. [file 12864_2022_8943_MOESM1_ESM.zip › bmc-suplymental.docx]

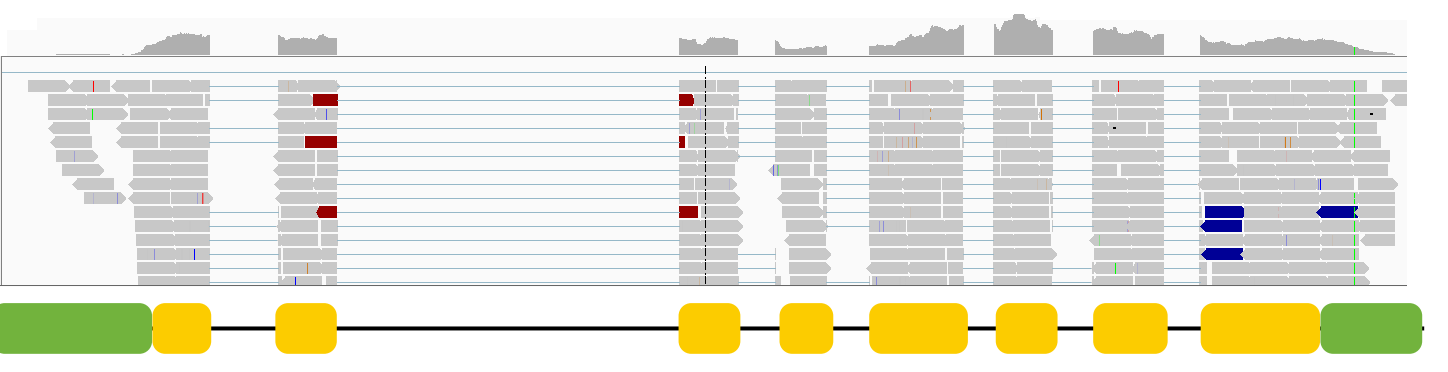


**Figure S1** Verification of *PpKO* gene structure using the alignment results of reads obtained from the transcriptome of peach flowers, stems, fruits and pits.


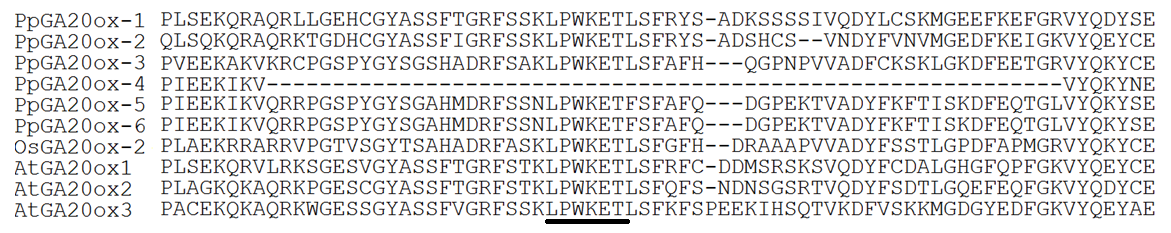


**Figure S2** Sequence alignment of GA20ox from Arabidopsis and peach. The LPWKET motif are underlined.


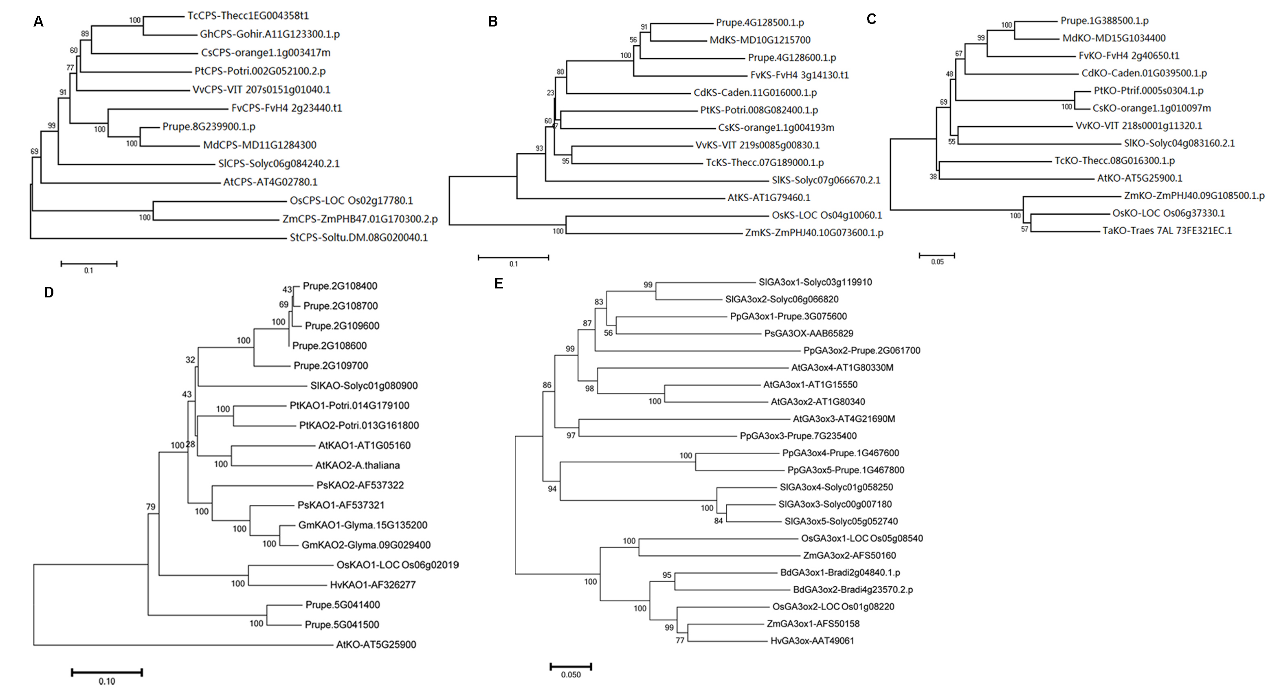


**Figure S3** The phylogenetic trees of CPS (A), KS (B), KO (C), KAO (D) and GA3ox (E). The amino acid sequences were downloaded from Phytozome database and the Gene ID are listed after the gene name.


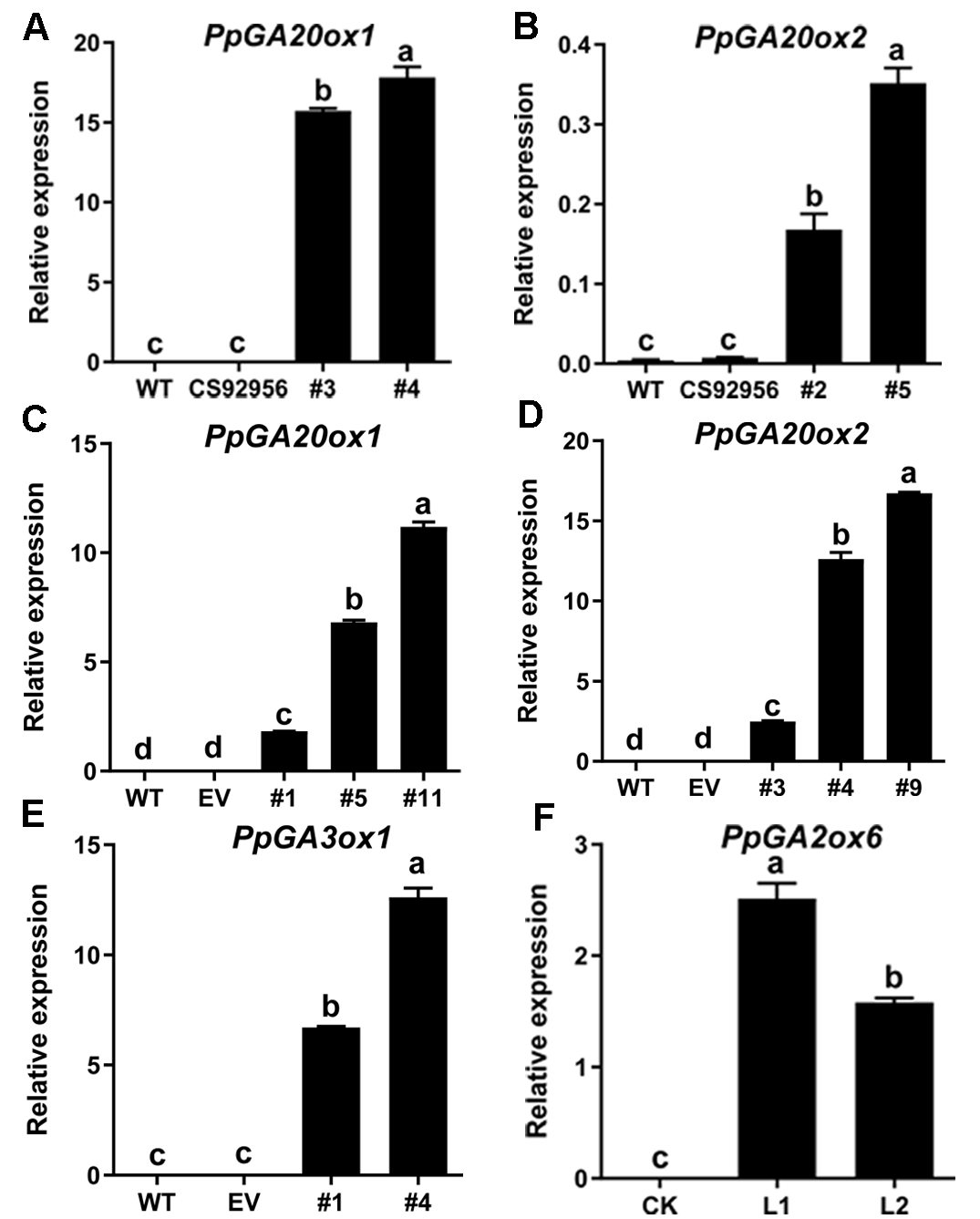


**Figure S4** qPCR analysis of transgenic lines. Two transgenic lines of *PpGA20ox1* (A) and -2 (B) in *ga20ox* mutant (CS92956) of Arabidopsis. Three transgenic lines of *PpGA20ox1* (C) and -2 (D) in Arabidopsis (Ecotype Columbia). Two lines of *PpGA3ox1* (E) in Arabidopsis (Ecotype Columbia). Two lines of *PpGA2ox6* in *Nicotiana tabacum*.


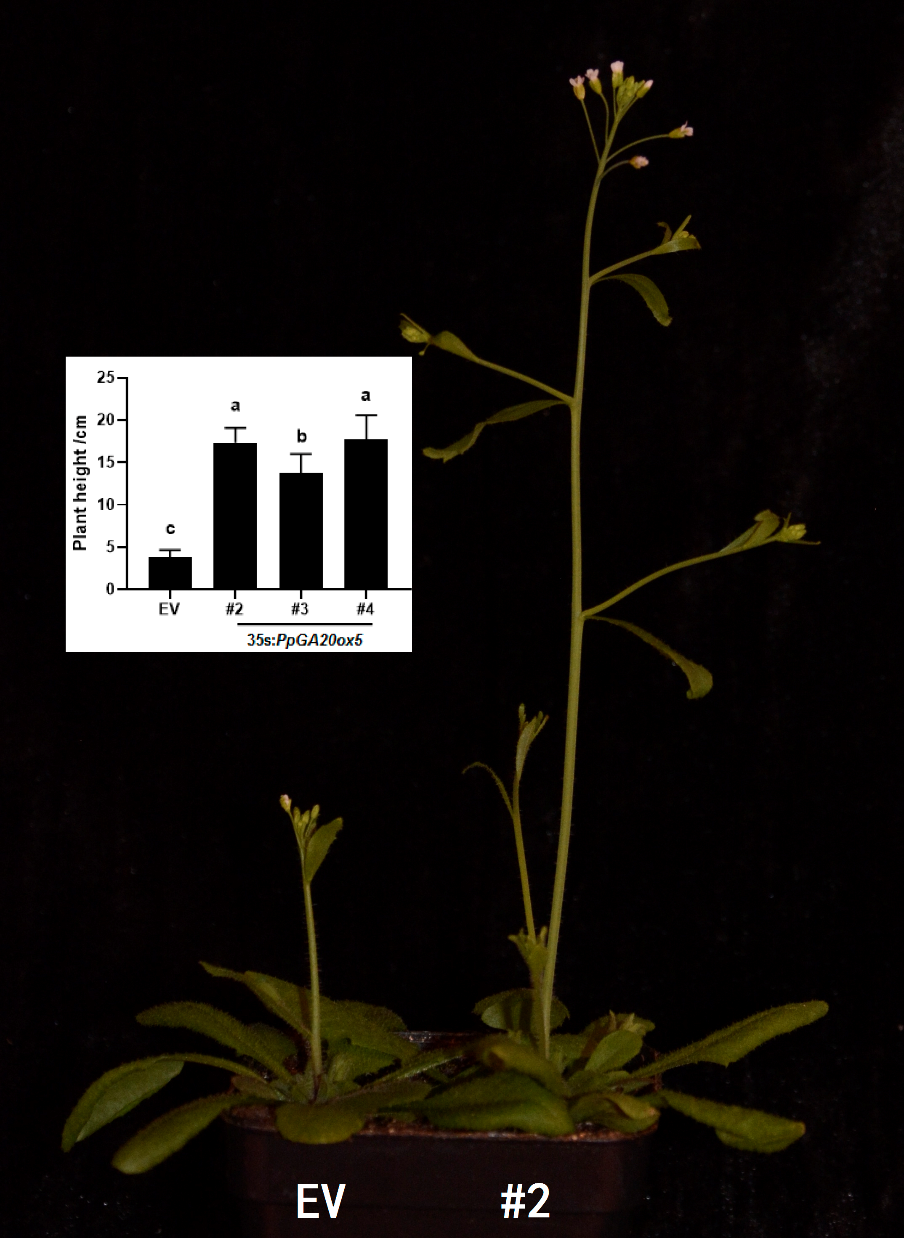


**Figure S5** Overexpression of *PpGA20ox5* in Arabidopsis.

**Table S2** **List of primers used in this study.**

| **Primer** | **sequence** |  |  |  |
| --- | --- | --- | --- | --- |
| **Primers for Real time PCR**   \| *CPS* \| 5’-CCTTCGGACGACATACACTCTT-3’/5’-GCACTGTGGCTAATCCTATGG-3’ \| \| --- \| --- \| \| *KS1*  *KS2* \| 5’-ACAGGGTCGTCACTGAGGAGG-3’/ 5’-GGCAACGATGGGTTCTTCAG-3’  5’- TTGAGAAACAAAATAACTGGATGAG-3’/5’-ATCTGTTCTCCACAACCCACC-3’ \| \| *KO*  *KAO1* \| 5’-CAATGGGAAAGCCCTGGAG-3’/ 5’-CTGAAGAGAACCAGCACAAACC-3’  5’-GAATCAAGAAAATTGGATCTGCA -3’/5’-TGCCAACCAAGAACTTAGAACC-3’ \| \| *KAO2*  *KAO3*  *KAO4*  *KAO5*  *KAO6*  *KAO7*  *PpGA20ox1*  *PpGA20ox2*  *PpGA20ox3*  *PpGA20ox5*  *PpGA20ox6*  *PpGA3ox1*  *PpGA3ox2*  *PpGA3ox3*  *PpGA3ox4*  *PpGA3ox5* \| 5’-GGCAAGAATCAAGAAAACTGGA-3’/5’-GCCAACCAAAAACATAGAACCT-3’  5’-GTCTGCCTCACATATTAGGTTTTAG -3’/5’- CCAAAACCAACTGCTCCTAAC-3’  5’- TATGAGAGGAGAAGTGAAGAAGGAA -3’/5’- AATGCACAATCACCAGCTCTTAT-3’  5’- TGCTGGTGATTGTGCATTTG-3’/5’-CAGCAACAAATACTAAAGGGCA-3’  5’-CTGGAAAGTAATGATGTG -3’/5’- GTCAAATCACTTCCAGGGCA-3’  5’- GATCAGTGGTCACAAGGCAT -3’/5’- ATTGGACCAGTCTCACAG-3’  5’-AACAGCCAAACACCAAGGAAGT-3’/5’-TCTTGGGCACGAAGTATCATCA-3’  5’-TGATTCCAAGAGCCCAAGG-3’/5’-ATCCAGTCTGAGAAGGCATCC-3’  5’-CAGAAGGAGAGGAGGTCAATGG-3’/5’-TTTGGAGGGTAGTTTCGTCAGG-3’  5’-GTGAGGGAGGCACAAGAAAGT-3’/5’- GAAAGGAACCAGTCGGTGAAGT-3’  5’-GTGAGGGAGGCACAAGAAAGT-3’/5’-GAAAGGAACCAGTCGGTGAAGT-3’  5’-GCAGACACCGTTTATCCATCG-3’/5’- GGACAGATGAAAGTGCCTTATTG-3’  5’-CGGTGACCTCATGCACATATT-3’/5’- AGGGTGGCTTGCGCTTAG-3’  5’-TTTCTATGCTCCTCCAGGTGAT-3’/5’-AAATGCTTGCCCTTGCTCT-3’  5’-CTCCTTTGCCTATTTCTATGCTC-3’/5’- TGCCCTTGATTCCAACATACT-3’  5’-GGATGTGAAGATATCGCCGTT-3’/5’- CTTGTTGAAGTGCGTTGCC-3’ \| \| *PpGA2ox1*  *PpGA2ox2*  *PpGA2ox3*  *PpGA2ox4*  *PpGA2ox5*  *PpGA2ox6*  *PpGA2ox7*  *PpGAPDH* \| 5’-TTTGATTGGTGAGGGATTGTG-3’/5’-GCTACTGTTGATGTTGCTGGTG-3’  5’-GATTCCAAATGGGTTGCTGTGA-3’/5’-AAGTAGGCGATTGAGTAGCGTTC-3’  5’-TCAAGGACCCATCACCAAAAC-3’/5’-AGGGACGGGAACCCACAA-3’  5’-ATCAAGACCAGGTTGGAGGACT-3’/5’-CTCATACGAAGGGCATAGGAAAT-3’  5’-GCATAGGGTTTTGGCAGACAC-3’/5’-GGACTTTTTGTATTCACACCACG-3’  5’-CTTGAACTGATGGCTGAAGGATT-3’/5’-GATGATTTGTGGGTCTGTGTGC-3’  5’-GCCTACTTCTACTGTCCTTCCTACG-3’/5’-AGTTCCTCATCTTTGGGCTTTGT-3’  5’-GACTTCATTGGTGACAGCAGGT-3’/5’-CGGGATTTAGAGAGTGGATGC -3’ \| | | |  |  |
| **Primer for vector construction** | | |  |  |
| *PpGA20ox1* | | 5’-CCG***CCCGGG***TGGCTTTTCTCTCATCACACTTC-3’/5’TGC***CTCGAG***TCCTCTAACTTTGCCTTTGTCC-3’ | | |
| *PpGA20ox2*  *PpGA20ox5*  *PpGA3ox1*  *PpGA2ox6* | | 5’-CGG***GGTACC***GTTCCAAAACTAAGAAGGCTGC-3’/5’-  CCG***CTCGAG***CTGGTGCTTCTTTCATGGTTAGT-3’  5’-CGG***GGTACC***CCGTGCCTCTCTCTCTCAATCT-3’/5’-  CCG***CTCGAG***CAGATGTAAATTATCAATGGGACGT-3’  5’- CGG***GGTACC***AAACACAAAACCCATCTTCTCTCTG -3’/5’- CCG***CTCGAG***ATTTATCTACCCGACTTTTACGCTG -3’  5’-CGGGGTACCACACGGAGGAGCAAGAACAACT-3’/5’-  CCGCTCGAGGGGAAGGGGGAAACAGAAGAG-3’ | | |
